# Primary retroperitoneal mucinous cystadenocarcinoma in a male patient: a case report

**DOI:** 10.4076/1757-1626-2-7196

**Published:** 2009-08-17

**Authors:** Abdelmalek Hrora, Sanae Reggoug, Houda Jallal, Farid Sabbah, Abdessalam Benamer, Mouna Alaoui, Mohamed Raiss, Mohamed Ahallat

**Affiliations:** Surgery Unit “Clinique Chirurgicale C”, University Hospital Ibn SinaRabatMorocco

## Abstract

In the literature, 51 cases of primary retroperitoneal mucinous cystadenocarcinoma have been published. We report the fourth case occurring in a male patient. The 42-year-old patient presented with multiple retroperitoneal cystic masses causing abdominal discomfort without alteration of the global clinical state. The masses were totally removed by a two-stage surgery. No other treatment has been introduced. After a follow-up of 6 months, the patient is disease-free. This rare tumor most likely arises from the mucinous metaplasia of peritoneal inclusion cysts rather than from ectopic ovarian tissue or ovarian teratomas. The occurrence of such a tumor in a male patient supports this theory. Preoperative diagnosis is mostly difficult. Clinical behavior and treatment are still controversial.

## Introduction

Primary retroperitoneal mucinous cystadenocarcinoma (PRMC) is an extremely rare tumor. To date, only 51 cases have been published. To our knowledge, this is the fourth case ever reported in a male patient. The pathogenesis remains unclear and controversial. Diagnosis can often be confusing preoperatively. Surgeons must be aware of this type of tumor and include it in their differential diagnosis. Due to its rarity, optimal treatment, survival and exact prognosis continue to be uncertain. We report a case of PRMC in a 42-year-old man. A two-stage surgical removal of all tumoral cysts has been performed.

## Case presentation

**Figure 1.**
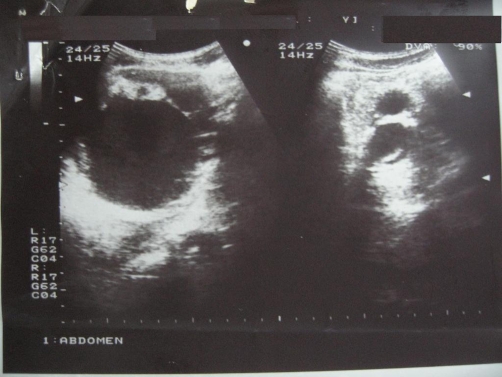
Abdominal ultrasound; cystic masses mimicking liver hydatid cysts.

A 42-year-old Moroccan man was initially seen in a private structure for complaints of abdominal discomfort and distension that lasted a year and a half. A notion of smoking weaned 4 years ago was found in his history. Prior physical examination found a large indolent right hypochondrium mass. Both testes were descended and were clinically normal. Biological tests, including Ca 19-9, were strictly normal. On abdominal ultrasonography (US), the mass was thought to be hydatid cyst of the liver (Figure 1). At prior laparotomy, liver appeared normal but five cystic and multilocular masses were discovered, in the median retroperitoneum, seated between inferior veina cava and the abdominal aorta bifurcation. Pancreas, spleen and kidneys were normal. Total excision was performed for 3 masses. The other two masses were inextirpable because of strong adherences to vessels. The patient was then referred to our surgical unit for complementary treatment.

**Figure 2.**
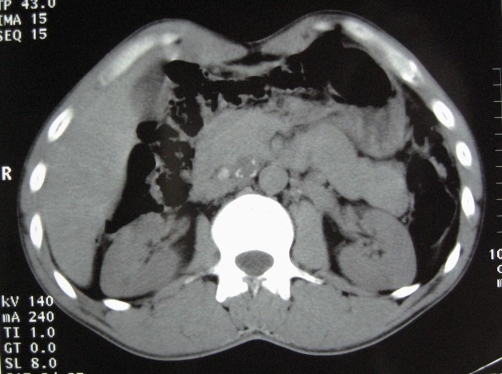
Computed tomography; retroperitoneal cystic mass, spontaneously hyperdense, containing calcifications.

**Figure 3.**
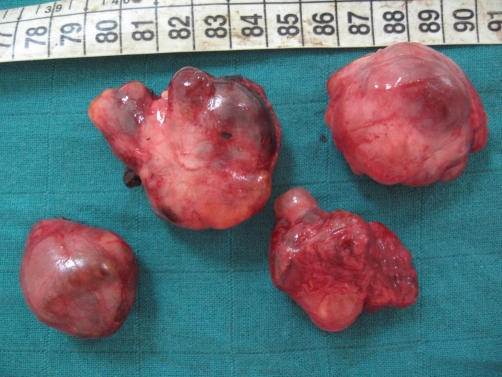
Macroscopic findings; four cystic masses, surgically removed, surrounded by a capsule with a network of small dilated vessels and without exophytic vegetations.

**Figure 4.**
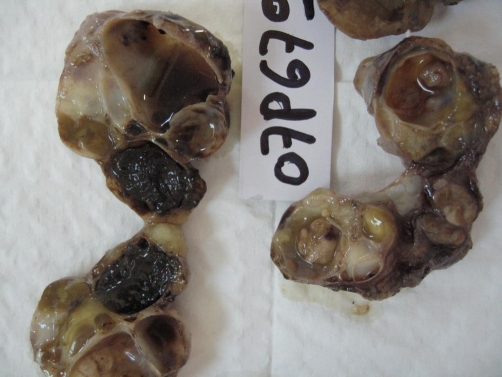
Macroscopic findings; multicystic aspect at sectioning, with mucoid content.

**Figure 5.**
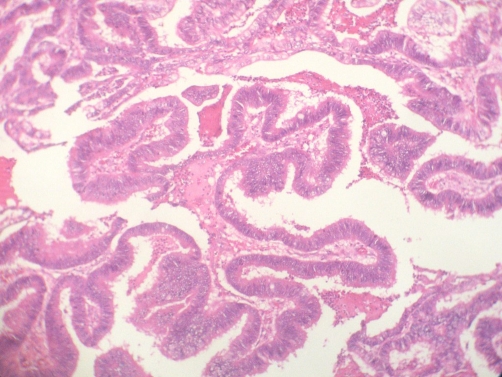
Microscopic findings; cystic wall was containing smooth muscle fibers and was lined by a monostratified mucinous type epithelium (hematoxilin and eosin, 20×). Papillary pseudostratification was also observed.

Computed tomography (CT) showed residual retroperitoneal masses with cystic and tissular components, spontaneously hyperdense (Figure 2). No enhancement was observed after injection. Magnetic resonance imaging (MRI) revealed multiple processes in the median retroperitoneum, extended from retro-cephalic pancreas to the aortic bifurcation and intimately adherent to retroperitoneal vessels without thrombosis. Hypersignal zones were observed on T1 and T2 with fat suppression suggesting a mucoid component. Some cysts showed carneous zones slightly enhanced after gadolinium injection. Furthermore, pancreas was homogenous with no visible nodular lesion. At the second laparotomy, 4 additional cystic masses were discovered in the retroperitoneum aligned from the duodenojejunal flexure to the aortic bifurcation. There was no clear evidence that the masses were originating from the pancreas or any other intra-abdominal organs. Cystic masses were totally removed. They appeared encapsulated, multicystic and filled with mucinous material (Figures 3-4). The largest one measured 5 × 4 × 3 cm. At histology, cystic wall contained some smooth muscle fibres and was lined by a monostratified epithelioma with mucin-producing columnar cells of the intestinal type (Figure 5). Pluristratified foci with papillary architecture and marked atypical cells were detected signalising a borderline malignancy. Fibro-inflammatory stroma was also noticed without invasive elements. This histological pattern was similar to that reported after the first surgical resection and suggested the diagnosis of PRMC. No complication occurred in the postoperative period and no adjuvant therapy has been instaured. There was no evidence of recurrence, radiologically attested, after 6 months of follow-up.

## Discussion

To date, only 51 cases of PRMC have been described. All were females [1-4] but three occurred in male patients [5-7]. We report the 52^th^ case and to our knowledge, it is the fourth male patient with PRMC published in the literature. The first male patient was reported by Motoyama et al. (1994), with little mention of clinical data and follow-up [6]. The second male patient, described by Thamboo et al. (2006) [7] presented with a large retroperitoneal cystic tumor measuring 24 × 20 × 16 cm, which was removed intact. The third one was reported by Green et al. (2007) with the largest tumor ever reported, measuring 26 × 21 × 16 cm on CT [5].

Pathogenesis of these tumors remains unknown. First hypothesis suggests that PRMCs arise from a retroperitoneal monodermal teratoma, with predominant columnar epithelium [8]. Other authors postulate an enterogenous genesis because of intestinal duplication [9]. The intestinal-like epithelioma and smooth muscles fibers surrounding the cystic tumors in our case could support this hypothesis. Because these tumors resemble ovarian mucinous cystic neoplasms, the third explanation is that they occur from ectopic ovarian tissue. Still no ovarian tissue has ever been found within a PRMC [1,2]. The fourth possibility is that the tumors are remnants of the embryonal urogenital apparatus, in which the cysts develop from specialized mesothelial cells of the urogenital ridge [5]. The most widely accepted theory is that PRMCs occur from invaginations of the peritoneal epithelium during embryonic growth and subsequently undergo metaplasia [10]. Moreover, PRMC could originate from undescended testis, what was excluded in our case.

Similarly to prior case reports, we were unable peroperatively to discover a pedicle or attachment to other organs. Histologically, these tumors can be perplexing because they present benign, borderline malignant and frankly malignant areas [6]. The previous reported PRMCs tended to be solitary and unilocular.

PRMCs often develop earlier in women with a mean age at presentation of 42.4 years (range: 17-86), whereas the diagnosis was assessed in the three previous male patients at 83, 63 and 64 years respectively [5-7]. Our patient was much younger with an age at onset of 42 years. The most common symptom is abdominal discomfort and a slow-growing pelvic or abdominal mass. In most cases, clinical course appears to be indolent. However, these tumors can become aggressive. Preoperative diagnosis is difficult because of non-specific symptoms and the scarce aid of imaging [11]. Tumor markers, such as CA-125 and CA19-9 are not very helpful due to their lake of specificity [12]. Differential diagnoses include metastatic mucinous tumors from sites such as ovaries, intestines (including the appendix) and pancreas. But also benign renal cystic disease, renal lymphangioma or hydatid cysts. Needle biopsy may be of use in these situations but is not a good measure to determine malignancy in cystic tumors.

Management of PRMC is not well established. Clearly, radical tumor excision is mandatory. Adjuvant chemotherapy has been attempted in some patients with limited success [2,13]. Chemotherapy should be limited to ruptured tumors during surgery [1,2] or when invasion to adjacent structures is evident. Given the assumption that PRMC arises in heterotopic ovarian tissue, many authors advise hysterectomy with bilateral salpingo-oophorectomy, either simultaneously or later at re-exploratory laparotomy [1,14]. In these cases, the resected genital organs showed no evidence of tumor infiltration and the follow-up of these patients is yet too short to allow valid conclusions to be drawn. Other authors recommend adjuvant hysterectomy and bilateral salpingo-oophorectomy restricted to women who have completed their child bearing or are postmenopausal [1].

Prognosis of these tumors remains uncertain because of their rarity and the short follow-up of the patients. The longest reported follow-up is 6 years in women [15] and 18 months in men [7]. Presence of a mural nodule within the cyst wall could worsen prognosis [13]. It is also difficult to determine whether these tumors have a similar clinical behavior and prognosis in men and women. None of the patients who died or received chemotherapy were men. Long-term follow-up after surgical removal of this unusual tumor with ultrasound or CT scanning appears thus essential.

## Conclusion

PMRC usually presents with mass effects, have an indolent course but can become very large or possess aggressive clinical course. Diagnosis remains difficult preoperatively and surgeons must be aware of PRMC as a differential diagnosis of a large retroperitoneal cystic mass. Clinical behavior and treatment of PRMC still are controversial. To date, extirpative surgery appears to be the standard treatment and the role of adjuvant radiation or chemotherapy has yet to be determined. Further studies are needed to establish optimal treatment protocols.

## Abbreviations

CT: computed tomography
MRI: magnetic resonance imaging
PRMC: primary retroperitoneal mucinous cystadenocarcinoma
US: ultrasonography
